# Neuroleptic Malignant Syndrome Induced by Multiple Antipsychotics in a Patient Receiving Methadone

**DOI:** 10.7759/cureus.101868

**Published:** 2026-01-19

**Authors:** Naoki Suzuki

**Affiliations:** 1 Palliative Care, Yamagata Prefectural Central Hospital, Yamagata, JPN

**Keywords:** antipsychotic side effect, methadone therapy, muscle rigidity, neuroleptic malignant syndrome (nms), palliative and end-of-life care

## Abstract

Neuroleptic malignant syndrome (NMS) is a rare but potentially life-threatening adverse reaction to dopamine antagonists. In palliative care settings, recognition of NMS may be difficult because delirium, polypharmacy, and metabolic disturbances are common, and atypical presentations have been reported. A 73-year-old woman with advanced gastric cancer was admitted for refractory cancer-related pain. Methadone was initiated, and multiple antipsychotics were subsequently prescribed for delirium. On hospital day 20, she developed altered mental status and generalized lead-pipe rigidity without fever or autonomic instability. Laboratory testing revealed leukocytosis and a moderate elevation of creatine kinase (422 U/L). Although she did not initially meet established diagnostic criteria for NMS, fever and tachycardia developed on the following day, supporting the diagnosis. Malignant catatonia was considered but deemed less likely based on clinical features and laboratory findings. All antipsychotics were discontinued, and the patient was managed conservatively without dantrolene or bromocriptine because of limited intravenous access and palliative goals of care. Her symptoms gradually resolved over several days. This case highlights the diagnostic challenges of atypical NMS in palliative care patients receiving multiple antipsychotics. Careful assessment of temporal symptom evolution and medication exposure is essential. In selected patients with advanced cancer, conservative management with withdrawal of causative agents and supportive care may be an effective treatment approach.

## Introduction

Neuroleptic malignant syndrome (NMS) is an uncommon yet serious adverse reaction to dopamine-blocking medications and is classically characterized by hyperthermia, muscle rigidity, altered mental status, and autonomic instability [1]. Although these diagnostic features are well described, atypical or incomplete presentations have long been reported, including cases with delayed onset of fever or without marked hyperthermia [2,3]. In patients with advanced cancer, NMS may be particularly challenging to recognize because delirium, infection, and metabolic abnormalities are common, while systematic data on its incidence in palliative care remain limited [4].

Patients receiving palliative care often undergo complex medication adjustments for pain, nausea, and delirium. Antipsychotics are frequently prescribed even for hypoactive delirium, as suggested by physician survey data [5]. This practice may lead to the introduction of multiple antipsychotics within short intervals, thereby increasing the risk of NMS and complicating the evaluation of emerging neurological symptoms. Opioids can further obscure the clinical picture. Methadone, in particular, has N-methyl-D-aspartate receptor antagonism and weak serotonergic properties and has been implicated in phenomena such as rigid chest syndrome and toxic leukoencephalopathy, which may mimic or overlap with features relevant to NMS [6-11].

Here, we present a patient with advanced cancer who developed NMS after the rapid introduction of multiple antipsychotics in the context of methadone therapy. The initial presentation lacked fever and clear autonomic instability, and creatine kinase elevation was moderate, which complicated the distinction from malignant catatonia and other differentials. We describe the diagnostic process and discuss the rationale for conservative management, aiming to offer practical guidance for the recognition and treatment of NMS in palliative care settings.

## Case presentation

A 73-year-old woman was admitted to the palliative care unit for severe neck and upper limb pain due to cervical spine metastasis from gastric cancer. She had undergone a distal gastrectomy two years earlier and subsequently received chemotherapy for lymph node recurrence. On admission, her vital signs were stable, she was alert and oriented, and no muscle rigidity or abnormal involuntary movements were observed. Pain control was the primary reason for hospitalization, and no acute neurological deficits were identified.

**Figure 1:**
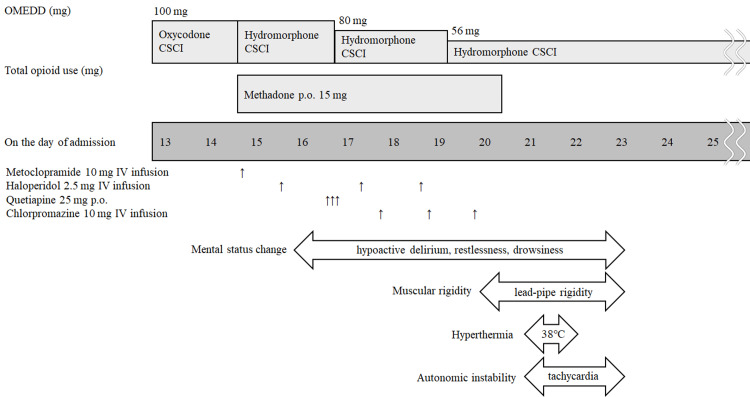
The patient’s clinical course and medication timeline The figure illustrates the progression of the patient’s clinical course, highlighting key events, interventions, and changes in symptoms over time. OMEDD, oral morphine equivalent daily dose; CSCI, continuous subcutaneous infusion; p.o., per os; IV, intravenous

The patient’s clinical course and medication timeline are summarized in Figure 1.

The patient had advanced cancer with poor functional status (Eastern Cooperative Oncology Group performance status 3) and markedly reduced oral intake, limited to a few mouthfuls per day [12]. Continuous subcutaneous oxycodone infusion was gradually escalated to 50 mg/day for pain control. Because analgesia remained inadequate, oral methadone was initiated at 15 mg/day on hospital day 14. On hospital day 15, she developed opioid-induced nausea and vomiting, for which haloperidol 2.5 mg was administered. On the following day, she exhibited impaired attention and disorientation and was diagnosed with delirium. Given the presence of hypoactive features, quetiapine was initiated at night. Despite these interventions, her mental status did not improve, and additional antipsychotics, including haloperidol and chlorpromazine, were introduced over a short period. Suspecting opioids as a potential contributor to delirium, oxycodone was switched to hydromorphone, and the total opioid dose was reduced. Benzodiazepines were not used at any time.

On the morning of hospital day 20, the patient showed a marked change in mental and physical status. She responded to verbal stimuli only by nodding and appeared somnolent but calm. Prominent generalized muscle rigidity was observed in all four limbs. Her upper extremities were crossed over the anterior chest and could not be extended either actively or passively, consistent with lead-pipe rigidity. No tremor, clonus, or involuntary movements were noted. Peripheral edema was present, making peripheral intravenous access difficult.

At that time, her vital signs showed no evidence of autonomic instability: body temperature was 36.4°C, heart rate 70 beats per minute, and blood pressure 100/67 mmHg. Respiratory rate was not formally recorded; however, there was no tachypnea or labored breathing on clinical observation. Oxygen saturation was adequately maintained on room air.

Laboratory findings at the time of symptom onset are summarized in Table 1.

**Table 1:** Laboratory and physical findings Reference ranges reflect those used by the testing laboratory at our institution. NMS, neuroleptic malignant syndrome

Variables	Reference ranges	Admission day	One week before NMS onset	NMS onset day	One day after NMS onset
White blood cell counts (/μL)	3300-8600	5580	7020	18,620	*
C-reactive protein (mg/dL)	0-0.14	*	0.29	16.7	*
Creatine kinase (U/L)	41-153	*	*	422	*
Myoglobin (ng/mL)	0-74	*	*	391	*
Total bilirubin (mg/dL)	0.4-1.5	0.4	0.5	0.7	*
Aspartate aminotransferase (U/L)	13-30	19	22	25	*
Alanine aminotransferase (U/L)	7-23	14	18	14	*
Lactate dehydrogenase (U/L)	124-222	263	350	276	*
Alkaline phosphatase (U/L)	38-113	107	136	131	*
Blood urea nitrogen (mg/dL)	8-20	21.7	22.7	29.1	*
Creatinine (mg/dL)	0.46-0.79	0.6	0.46	0.61	*
Sodium (mEq/L)	138-145	140	127	124	*
Potassium (mEq/L)	3.6-4.8	3.6	5.0	4.0	*
Corrected calcium (mg/dL)	8.8-10.1	9.8	*	7.7	*
Phosphate (mg/dL)	2.7-4.6	*	*	1.6	*
Magnesium (mg/dL)	1.8-2.3	*	*	2.3	*
Body temperature (℃)	*	36.7	36.4	36.4	38.0
Heart rate (/min)	*	72	80	70	122
Blood pressure (mmHg)	*	149/93	117/71	100/67	103/68

Laboratory evaluation on hospital day 20 revealed leukocytosis and biochemical abnormalities. The white blood cell count was elevated to 18,620/μL, serum creatine kinase to 422 U/L, and serum myoglobin to 391 ng/mL. C-reactive protein was elevated to 16.7 mg/dL. Electrolyte abnormalities were present, including hyponatremia (serum sodium, 124 mEq/L), which was considered primarily related to poor oral intake. Liver enzyme levels were within normal limits. Compared with earlier results, creatine kinase and inflammatory markers showed a clear upward trend, whereas electrolyte disturbances had preceded the onset of muscle rigidity. No imaging studies or cerebrospinal fluid examination were performed because the patient’s advanced disease status and goals of care favored a symptom-oriented approach.

At the time muscle rigidity was first recognized, the patient did not fully meet the diagnostic criteria for NMS according to the Diagnostic and Statistical Manual of Mental Disorders, Fifth Edition, Text Revision, Caroff’s criteria, or Levenson’s criteria [2,3,13]. Although marked rigidity and altered mental status were present, she was afebrile and showed no clear signs of autonomic instability. Malignant catatonia was therefore considered in the differential diagnosis; however, the absence of a preceding psychotic state, the lack of psychomotor excitement or stupor, and the presence of leukocytosis and elevated creatine kinase favored NMS. Benzodiazepines were not administered, and no catatonia-specific diagnostic challenge was performed.

On the following day, the patient developed fever and tachycardia, fulfilling the remaining diagnostic components required for NMS. The temporal association between the rapid introduction of multiple antipsychotics and the subsequent evolution of neuromuscular and systemic findings further supported this diagnosis.

All antipsychotic medications were discontinued on hospital day 20. Methadone was also stopped on the same day because of the patient’s difficulty with oral intake; the final dose had been administered after the onset of muscle rigidity. Because peripheral edema prevented intravenous access and injectable methadone is not available in Japan, treatment options were limited. Given the patient’s advanced cancer, poor functional status, and goals of care, aggressive interventions such as intravenous fluid resuscitation or pharmacologic treatment with dantrolene or bromocriptine were not pursued. Instead, the patient was managed conservatively with close clinical observation and supportive care.

Her level of consciousness gradually improved, and muscle rigidity began to resolve on hospital day 21. By hospital day 22, rigidity had further improved, and by hospital day 23, it had completely resolved. After resolution of NMS, low-dose risperidone (0.5 mg) was administered intermittently on an as-needed basis for nighttime delirium without recurrence of neuromuscular or autonomic abnormalities. Although NMS resolved, the patient’s overall condition gradually deteriorated because of progressive cancer, and she died on hospital day 36. No autopsy was performed.

## Discussion

This case highlights several important considerations regarding the diagnosis and management of NMS in a palliative care setting. First, the clinical presentation was atypical, with generalized rigidity and altered mental status preceding the development of fever and autonomic instability. Second, the patient improved with conservative management alone, without the use of specific pharmacologic therapies such as dantrolene or bromocriptine. These features are particularly noteworthy in the context of advanced cancer, where polypharmacy is common, and baseline vulnerability often obscures the recognition of serious adverse drug reactions, despite previous reports of NMS in non-cancer populations.

The diagnosis of NMS in this patient was challenging because she did not initially fulfill established diagnostic criteria. On hospital day 20, marked rigidity and altered mental status were present, but fever and clear autonomic instability were absent. Delayed onset of hyperthermia has been described in early reports of NMS, and afebrile or incomplete presentations are well recognized, particularly in medically complex patients [2,3]. In the present case, fever and tachycardia developed on the following day, completing the diagnostic picture. This temporal evolution underscores the importance of repeated clinical assessment rather than reliance on a single time point when evaluating suspected NMS, especially in palliative care patients. Serotonin syndrome was also considered in the differential diagnosis, particularly given the weak serotonergic properties of methadone. However, this was considered unlikely due to the absence of hyperreflexia, clonus, gastrointestinal symptoms, and rapid symptom onset, as well as the presence of lead-pipe rigidity and marked creatine kinase elevation, which are more consistent with NMS.

Malignant catatonia was an important differential diagnosis. Both conditions share overlapping features, including rigidity, altered consciousness, and autonomic disturbances. However, several aspects favored NMS in this case. There was no history of a primary psychotic disorder, no preceding psychomotor excitement or stupor, and no exposure to benzodiazepines that might have clarified catatonia through a therapeutic response. In contrast, the patient had been exposed to multiple dopamine antagonists over a short period, and laboratory findings showed leukocytosis and creatine kinase elevation, which are more characteristic of NMS. Although catatonia-specific diagnostic challenges were not performed, the overall clinical course and response to withdrawal of antipsychotics supported NMS as the most consistent diagnosis.

Polypharmacy played a central role in this case. In palliative care, antipsychotics are frequently prescribed for delirium, including hypoactive subtypes, and may be added or switched rapidly in response to fluctuating symptoms [1,5,14,15]. This practice increases the risk of cumulative dopamine blockade and may delay recognition of adverse effects. In addition, opioids can further complicate neurological assessment. Methadone, while not considered a direct cause of NMS, has pharmacological properties, including N-methyl-D-aspartate receptor antagonism and weak serotonergic activity, that may modify neuromuscular tone or mental status [16,17]. Reports of methadone-associated rigid chest syndrome and toxic leukoencephalopathy suggest that methadone can mimic or overlap with features relevant to NMS [10,11]. In the present case, methadone was unlikely to be the primary trigger because the final dose was administered after the onset of rigidity; however, its presence may have contributed to diagnostic uncertainty. This highlights the need for careful temporal analysis when multiple centrally acting drugs are used concurrently.

Another important aspect of this case is the successful use of conservative management. Standard recommendations for NMS emphasize immediate withdrawal of offending agents, supportive care, and consideration of dantrolene or dopaminergic agents in moderate to severe cases [1]. In this patient, intravenous access could not be established because of marked edema, and the goals of care prioritized comfort over aggressive intervention. Despite these limitations, withdrawal of antipsychotics and close observation resulted in gradual resolution of rigidity and systemic symptoms. This outcome suggests that, in selected palliative care patients with mild to moderate laboratory abnormalities and stable vital signs, conservative management may be a reasonable option. Such decisions must be individualized, taking into account disease stage, functional status, and patient-centered goals.

This case has several limitations. It represents a single observation, and causality between specific medications and symptom development cannot be definitively established. Extensive diagnostic testing was not performed, and benzodiazepine responsiveness was not assessed. Nonetheless, the case provides practical insight into the diagnostic complexity of NMS in advanced cancer patients and emphasizes the importance of vigilance when antipsychotics are used in combination. Early recognition of atypical presentations and timely withdrawal of causative agents remain critical, even when classical features are initially absent.

## Conclusions

This case illustrates that NMS can present with atypical features, including delayed fever and moderate creatine kinase elevation, in palliative care patients receiving multiple antipsychotics. Careful attention to temporal changes and medication exposure is essential to distinguish NMS from malignant catatonia and other mimicking conditions. In selected patients with advanced cancer, conservative management based on withdrawal of causative agents and supportive care may be an appropriate and effective treatment strategy.
